# Endothelin Receptor A Antagonism Attenuates Renal Medullary Blood Flow Impairment in Endotoxemic Pigs

**DOI:** 10.1371/journal.pone.0021534

**Published:** 2011-07-08

**Authors:** Johan Fenhammar, Andreas Andersson, Jakob Forestier, Eddie Weitzberg, Alf Sollevi, Hans Hjelmqvist, Robert Frithiof

**Affiliations:** 1 Department of Anaesthesiology & Intensive Care, Karolinska University Hospital Huddinge, and Department for Clinical Science Intervention and Technology, Karolinska Institutet, Huddinge, Stockholm, Sweden; 2 Department of Physiology & Pharmacology, Section for Anaesthesiology and Intensive Care, Karolinska Institutet, Stockholm, Sweden; 3 Department of Physiology & Pharmacology, Karolinska Institutet, Stockholm, Sweden; Universidade de Sao Paulo, Brazil

## Abstract

**Background:**

Endothelin-1 is a potent endogenous vasoconstrictor that contributes to renal microcirculatory impairment during endotoxemia and sepsis. Here we investigated if the renal circulatory and metabolic effects of endothelin during endotoxemia are mediated through activation of endothelin-A receptors.

**Methods and Findings:**

A randomized experimental study was performed with anesthetized and mechanically ventilated pigs subjected to Escherichia coli endotoxin infusion for five hours. After two hours the animals were treated with the selective endothelin receptor type A antagonist TBC 3711 (2 mg⋅kg^−1^, n = 8) or served as endotoxin-treated controls (n = 8). Renal artery blood flow, diuresis and creatinine clearance decreased in response to endotoxemia. Perfusion in the cortex, as measured by laser doppler flowmetry, was reduced in both groups, but TBC 3711 attenuated the decrease in the medulla (p = 0.002). Compared to control, TBC 3711 reduced renal oxygen extraction as well as cortical and medullary lactate/pyruvate ratios (p<0.05) measured by microdialysis. Furthermore, TBC 3711 attenuated the decline in renal cortical interstitial glucose levels (p = 0.02) and increased medullary pyruvate levels (p = 0.03). Decreased creatinine clearance and oliguria were present in both groups without any significant difference.

**Conclusions:**

These results suggest that endothelin released during endotoxemia acts via endothelin A receptors to impair renal medullary blood flow causing ischemia. Reduced renal oxygen extraction and cortical levels of lactate by TBC 3711, without effects on cortical blood flow, further suggest additional metabolic effects of endothelin type A receptor activation in this model of endotoxin induced acute kidney injury.

## Introduction

Sepsis is a common cause of acute kidney injury (AKI). The pathophysiology of septic AKI remains elusive but renal impairment during sepsis has been postulated to be mediated by kidney hypoperfusion owing to excessive renal vasoconstriction [1]. Endothelin-1 (ET-1) was first described by Yanagisawa and co-workers in 1988 and is a very potent endogenous vasoconstrictor [2]. Plasma levels of ET-1 is elevated in septic patients, mainly through increased production, and also associated with severity of the illness [3]. ET-1 mediates its effects through two different receptors, endothelin type A receptor (ETA) and endothelin type B receptor (ETB). In animal experiments of endotoxemia dual ETA/ETB endothelin blockade has been described to improve cardiopulmonary function [4], reduce pulmonary hypertension [5] and lung injury [6], attenuate intestinal microcirculatory dysfunction [7], reduce intestinal acidosis [8], [9] and improve renal function [10]. In the kidney, ET-1 may affect renal microcirculation by constricting afferent and efferent arterioles through activation of ETA [11]. Furthermore, ET-1 has tubular effects favoring diuresis and natriuresis. This may be mediated by a reduction of Na/K ATPase activity in the collecting ducts [12] and also through an inhibitory effect on the opening of epithelial Na channels by ETB [13]. A diuretic effect of ET-1 is suggested to primarily be due to inhibition of arginine-vasopressin stimulated water permeability by activation of ETB [14]. In addition, in rats with diabetic renal injury selective ETA-antagonism have been reported to reduce macrophage infiltration, suggesting that ETA activation also contributes to renal inflammation [15].

We have previously shown that treatment with tezosentan, a dual ETA/ETB antagonist, improves renal artery blood flow and renal cortical microcirculation in endotoxemia, but has no effect on urine production [16]. In the present study we hypothesized that endothelin mediates renal microcirculatory impairment in endotoxemia through activation of ETA and that treatment with TBC 3711, a selective ETA antagonist, would improve renal microcirculation and function. The theoretical rationale for this was that the diuretic effects of ETB would be preserved whereas the vasoconstrictive and pro-inflammatory properties of ETA would be inhibited.

## Materials and Methods

The experiments were conducted in accordance with “The European Convention for Protection of Vertebrate Animals used for Experimental and other Scientific Purposes” (Council of Europe No 123, Strasbourg 1985). The study protocol was approved by the Stockholm South Regional Ethics Committee for Experiments in Animals, Huddinge District Court, Stockholm, Sweden (Approval ID S-192-06). Sixteen crossbred (Landrace/Yorkshire/Hampshire) female pigs weighing 33.3±0.7 kg were used. Animals were fasted for solid food but had free access to water for 24 h before surgery.

### Surgical preparation

Anesthesia was initiated and maintained as previously described in detail [16]. Anesthesia during surgical preparation was maintained with sevoflurane (2.6% end-tidal concentration (Et%), followed by 1.0 Et% throughout the experiment). Fentanyl (10 µg⋅kg^−1^⋅h^−1^) and midazolam (0.15 mg⋅kg^−1^⋅h^−1^) was infused throughout the experiment as a complement to sevoflurane and to reduce surgically induced pain. Pancuronium bromide (0.5 mg⋅kg^−1^⋅h^−1^) was given for muscle paralysis. After oral intubation, ventilation with oxygen in air (FiO2 0.44) and a PEEP of 4 cm H_2_O was initiated. Ventilation was adjusted to reach a *P*CO_2_ of 4.7–5.3 kPa at baseline and the ventilator settings were then kept constant throughout the experiment. During the surgical preparation hydroxyethyl starch (130/0.4, 60 mg/ml, Voluven, Fresenius Kabi, Uppsala, Sweden) and saline with glucose (25 mg⋅ml^−1^) was infused continuously at 10 ml⋅kg^−1^⋅h^−1^ and 20 ml⋅kg^−1^⋅h^−1^, respectively.

Animals were placed in a supine position and the left carotid artery was cannulated with a single lumen catheter for measurement of arterial pressure and blood sampling. The jugular vein on the left side was also catheterized and used for fluid administration. A balloon-tipped pulmonary artery catheter (7.5F Swan-Ganz, Edwards Lifesciences, Irvine, CA) was inserted into the pulmonary artery via an introducer in the right jugular vein. The position in the pulmonary artery was determined by pressure guidance on a monitor. The different blood pressures were measured via pressure transducers (DPT-6003, PVB Medizin Technik, BMBH, Kirchseen, Germany). The pressure transducers were calibrated to atmospheric pressure at the level of the heart and to 100 mmHg or 25 mmHg using a saline column. After a midline laparotomy, an ultrasonic flow probe (Transonic Systems Inc, Ithaca, NY), was placed around the left renal artery and connected to a recorder (T206, Transonic Systems Inc). The left renal vein was visualized and an 18 Gauge catheter was inserted into the vein and carefully fixated. The urinary bladder was catheterized with a Foley catheter for hourly urine collection. The left kidney was then exposed and a laser Doppler probe (0.25 mm fiber separation, 780 nm wavelength; Perimed AB, Järfälla, Sweden) was sutured to the surface for cortical measurements. A needle laser Doppler probe (0.15 mm fiber separation, 780 nm wavelength) was then inserted 10–12 mm into the kidney for medullary measurements. Position was confirmed visually by opening the kidney post mortem. The two probes were connected to a Periflux 5001 base unit (780 nm wavelength, 15 kHz band width, 0.2-s time constant; Perimed AB). Calibration was performed according to the manufacturer's instructions. Two microdialysis catheters (CMA 20, membrane length 10 mm, shaft length 14 mm, diameter 0.5 mm, 20,000 Dalton membrane cut-off, CMA Microdialysis, Stockholm, Sweden) was inserted in the left kidney. First a small opening of the capsule was made with a needle, thereafter one probe was inserted in the cortical region of the kidney. The second microdialysis catheter was inserted in a right angle in a separate opening of the capsule and placed in the renal medulla. Position was confirmed visually by opening the kidney post mortem. The two probes were continuously perfused (CMA 402 syringe pump, CMA Microdialysis) at a speed of 1 µl⋅min^−1^ with a perfusion solution (T1 solution, 147 mM Na^+^, 4 mM K^+^, 2.3 mM Ca^2+^, and 156 mM Cl^−^, CMA Microdialysis). A stabilization period of 60 minutes was allowed before baseline measurements. Samples were collected for 10 minutes at baseline, 120 and 300 minutes after start of endotoxemia. Adjustment for time delay of samples reaching the vials was done. All samples were analyzed immediately on a bench top analyzer (CMA 600 microdialysis analyzer, CMA Microdialysis). After surgical preparation, the abdomen was carefully closed and the animals were allowed 60 minutes of recovery before the initiation of the experimental protocol. A continuous infusion of Ringeŕs Acetate (15 ml⋅kg^−1^⋅h^−1^) and saline with glucose 25 mg⋅ml^−1^ (5 ml⋅kg^−1^⋅h^−1^) was started directly after the surgery and kept constant throughout the experiment.

### Hemodynamic measurements and calculations

Hemodynamic measurements were acquired online (MP150, Biopac Systems, Goleta, CA) with acquisition software (AcqKnowledge 3.7.3.; Biopac Systems) and stored on a computer. Microcirculatory measurements were recorded online with Perisoft for Windows (Perimed AB) data acquisition software. Cardiac output was indexed to body surface area [17] and presented as a cardiac index (CI). Renal artery blood flow (RBF) was indexed to bodyweight. Creatinine clearance was calculated as [(Urine flow × Urine creatinine concentration)/plasma creatinine concentration]. Renal vascular conductance was calculated as RBF divided by the difference of MAP and renal venous pressure. Oxygen delivery, oxygen consumption and oxygen extraction ratio was calculated according to standard formulas for both systemic and renal measurements.

### Blood and plasma samples

Blood samples were collected in pre-chilled EDTA tubes and immediately centrifuged at 3000 r.p.m. (200 g) for 15 min at 4°C to obtain plasma. The plasma was stored at 80°C until assayed. Plasma creatinine concentration was analyzed by the Jaffe method (Synchron LX, Beckman Instruments, Richmond, CA). The carotid blood samples were used for immediate arterial blood gas analyses (ABL 77, Radiometer, Copenhagen, Denmark). In addition, lactate (Accu-trend Lactate, Roche Diagnostics, Basel, Switzerland) were analyzed using arterial blood. Renal vein samples were analyzed for blood gases and lactate at baseline, 120 and 300 minutes after the infusion of endotoxin was initiated. ET-1 levels in arterial blood and renal vein was measured by radioimmunoassay as described earlier by Hemsen [18].

### Experimental protocol

Baseline data were collected immediately prior to endotoxemia. After baseline measurements all animals received a continuous infusion of endotoxin (Escherichia coli lipopolysaccharide, serotype 0111:B4, 900 000 units⋅mg^−1^ endotoxin, Sigma-Aldrich Sweden AB, Stockholm, Sweden). Endotoxin infusion was started at 0.3125 µg ⋅kg^−1^⋅h^−1^ and was increased stepwise until reaching 2.5 µg⋅kg^−1^⋅h^−1^ after 30 min. It was then kept constant throughout the experiment. After 120 minutes of endotoxemia animals were randomized to receive treatment with the ETA antagonist TBC 3711 (10 mg⋅ml^−1^, Encysive Pharmaceuticals Inc., Houston, TX,) 2 mg⋅kg^−1^ (n = 8) or no treatment (endotoxin-treated control, n = 8). The dose of TBC 3711, not having any ETB effect, was based on the results of an earlier study by our group [7]. At the end of the experiment the animals were deeply anesthetized and sacrificed by a lethal dose of sodium pentobarbital injected into a central vein.

### Statistical analysis

All statistical calculations were performed using Statistica 8.0 (Statsoft Inc., Tulsa, OK) and the graphs were created with Sigma Plot 11.0 (SPSS Inc., Chicago, IL). Data are expressed as means ± standard error of the mean (SEM). Changes in parameters over time were analysed according to a two-way repeated measures ANOVA. Main effects (time and treatment) were analyzed with time as a repeating variable before intervention, which included the time-points baseline, 60 and 120 minutes, and after intervention, which included the time-points 120, 180, 240 and 300 minutes, as within effects and treatment (control/TBC 3711) as between effects. A significant interaction between time and treatment was interpreted as a difference in the response to E.coli LPS over time between the groups. The significance level was set at *p*≤0.05.

## Results

### Effect of endotoxin, (Baseline-120)

#### Systemic parameters

**Figure 1:**
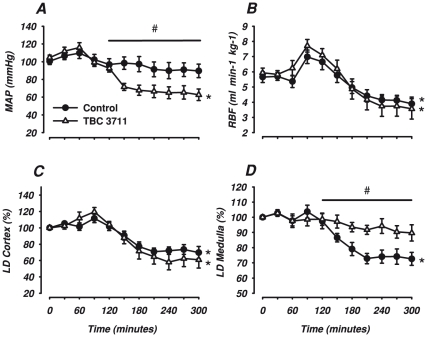
Renal hemodynamics. Changes in mean arterial blood pressure (MAP) (A), renal blood flow (RBF) (B), Laser Doppler flowmetry renal cortex (LD Cortex) (C) and Laser Doppler flowmetry renal medulla (LD Medulla) (D) in response to lipopolysaccharide infusion and treatment with either the selective ETA antagonist, TBC 3711 (2 mg·kg^−1^, n = 8) or control (n = 8). Data are expressed as mean ± SEM. # indicates a significant difference between TBC 3711 and control in response to lipopolysaccharide. (ANOVA repeated measures including 120, 180, 240 and 300). * indicates a significant change in effect over time (120–300).

Infusion of endotoxin reduced cardiac output, pH and increased heart rate, body temperature and arterial lactate (p<0.001, for all, Table 1). Endotoxemia had no significant effect on blood pressure (Figure 1). ET-1 levels in plasma increased significantly (p<0.001, Table 1). Systemic oxygen delivery, DO2_sys_ and ERO2_sys_ showed no significant change but systemic oxygen uptake, VO2_sys_ increased and base excess decreased (data not shown).

**Table 1:** Systemic and renal parameters.

	Group	Baseline		T60		T120		T180		T240		T300		p-value
		Mean	SEM	Mean	SEM	Mean	SEM	Mean	SEM	Mean	SEM	Mean	SEM	
Cardiac Index	Control	6,4	0,3	4,6	0,3	5,1	0,2	3,0	0,1	2,6	0,2	2,8	0,2	
(l/min/m^2^)	TBC	6,4	0,3	5,3	0,3	4,9	0,3	3,3	0,3	2,7	0,3	3,0	0,3	0,67
Heart rate	Control	100	4	115	8	136	6	148	9	169	10	178	8	
(beats•min^−1^)	TBC	100	5	121	7	150	9	160	11	171	12	168	13	0,09
Temperature	Control	36,9	0,2	37,4	0,2	38,1	0,1	38,4	0,1	38,7	0,1	38,8	0,1	
(°C)	TBC	37,1	0,2	37,6	0,2	38,2	0,2	38,6	0,3	38,9	0,2	39,0	0,2	0,95
Lactate_arterial_	Control	1,3	0,1	1,4	0,1	1,7	0,2	2,4	0,1	2,8	0,2	2,9	0,3	
(mmol/l)	TBC	1,2	0,1	1,3	0,1	2,0	0,2	2,5	0,2	2,8	0,3	2,5	0,2	0,21
pH	Control	7,47	0,01	7,44	0,01	7,43	0,01	7,40	0,02	7,34	0,02	7,30	0,03	
	TBC	7,47	0,00	7,46	0,01	7,43	0,01	7,40	0,01	7,37	0,02	7,37	0,02	0,01
Plasma- ET-1	Control	7,5	0,5	9,0	1,4	20,0	0,9	17,7	1,1	19,9	2,4	16,9	0,8	
(pmol/l)	TBC	7,0	0,9	9,0	0,9	21,7	1,5	23,4	2,2	23,7	3,1	18,5	1,7	0,77
Renal_venous_ ET-1	Control	4,7	0,4	-	-	13,5	2,7	-	-	-	-	10,7	2,1	
(pmol/l)	TBC	5,0	0,4	-	-	10,8	1,9	-	-	-	-	13,1	3,1	0,06
RVC	Control	2,1	0,1	1,8	0,1	2,6	0,2	2,0	0,1	1,8	0,1	1,6	0,1	
(min•ml^−1^•mmHg^−1^)	TBC	2,1	0,1	2,0	0,2	2,9	0,2	2,8	0,3	2,2	0,2	2,1	0,3	0,28

Data are for TBC 3711 (TBC, n = 8) and Control (n = 8) expressed as mean ± SEM. ANOVA repeated measures including 120, 180, 240 and 300 was performed. The p-value refers to the time-treatment interaction using these time-points. Differences were considered significant at *p*≤0.05. ET-1 = Endothelin-1, RVC = Renal vascular conductance,.

#### Renal parameters

**Figure 2:**
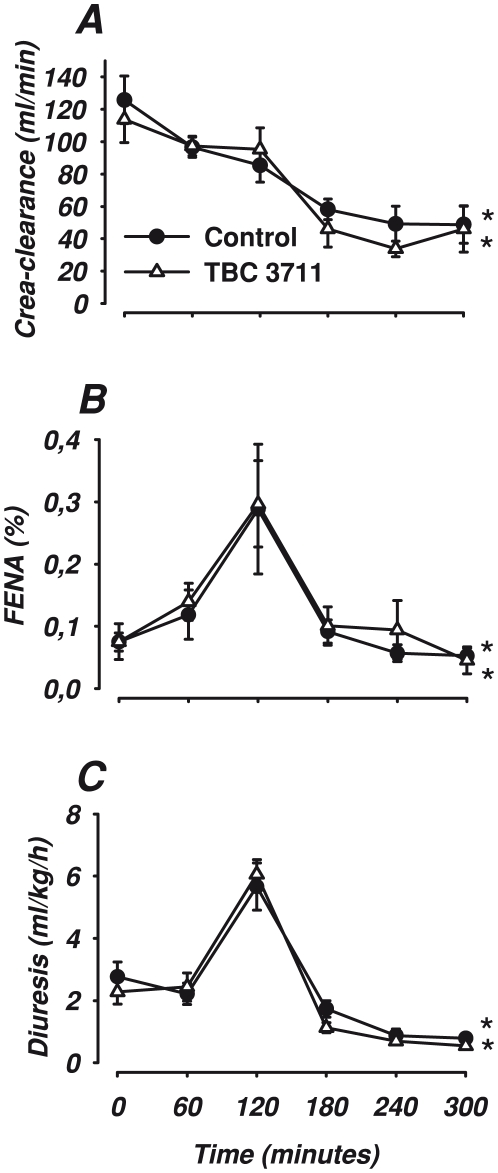
Renal function parameters. Changes in Creatinine clearance (A), Fractional excretion of sodium (FENA) (B) and diuresis (C) in response to lipopolysaccharide infusion and treatment with either the selective ETA antagonist, TBC 3711 (2 mg·kg ^−1^, n = 8) or control (n = 8) Data are expressed as mean ± SEM. **#** indicates a significant difference between TBC 3711 and control in response to lipopolysaccharide. (ANOVA repeated measures including 120, 180, 240 and 300). * indicates a significant change in effect over time (120–300).

Renal blood flow and renal vascular conductance increased significantly during the first two hours (p<0.01, Figure 1 and p<0.001, Table 1). Endotoxemia also resulted in an increased diuresis, fractional sodium excretion, FE_Na_ (p<0.001, for both, Figure 2) and renal vein lactate (p<0.001, Table 2). Laser Doppler flowmetry in renal cortex and medulla did not change significantly for the first 120 minutes (Figure 1), nor did creatinine clearance (Figure 2).

**Table 2:** Renal oxygen transport and metabolism.

	Group	Baseline		120		300		p-value
		Mean	SEM	Mean	SEM	Mean	SEM	
DO2_renal_	Control	19,5	0,9	31,3	3,0	18,0	2,3	
(ml•min^−1^)	TBC	20,9	1,1	35,2	1,9	16,8	3,0	0,10
Lactate_rena-v_	Control	1,4	0,1	2,1	0,1	3,4	0,3	
(mmol/1)	TBC	1,5	0,1	2,2	0,2	2,8	0,2	0,08
ISF-Glucose_cort_	Control	2,1	0,22	1,3	0,16	0,4	0,15	
(mmol/l)	TBC	2,2	0,17	1,5	0,13	1,4	0,17	0,02
ISF-Lactate_cort_	Control	0,5	0,09	0,8	0,13	1,7	0,31	
(mmol/1)	TBC	0,4	0,04	0,8	0,05	1,1	0,12	0,03
ISF-Pyruvate_cort_	Control	57	6	73	11	75	14	
(µmol/l)	TBC	41	4	69	7	99	5	0,05
ISF-Glucose_med_	Control	2,8	0,50	2,0	0,48	1,3	0,54	
(mmol/l)	TBC	2,8	0,32	2,5	0,29	2,1	0,48	0,27
ISF-Lactate_med_	Control	0,7	0,10	1,3	0,16	2,2	0,18	
(mmol/l)	TBC	0,9	0,05	1,2	0,14	2,2	0,28	0,93
ISF-Pyruvate_med_	Control	93	12	112	10	93	11	
(µmol/l)	TBC	98	16	125	17	159	24	0,01

Data are for TBC 3711 (TBC, n = 8) and Control (n = 8) expressed as mean ± SEM. ANOVA repeated measures including 120 and 300 was performed. The p-value refers to the time-treatment interaction using these time-points. Differences were considered significant at *p*≤0.05. cort = cortex, DO2renal = Renal oxygen delivery, ISF = Interstitial fluid, med = medulla.

**Figure 3:**
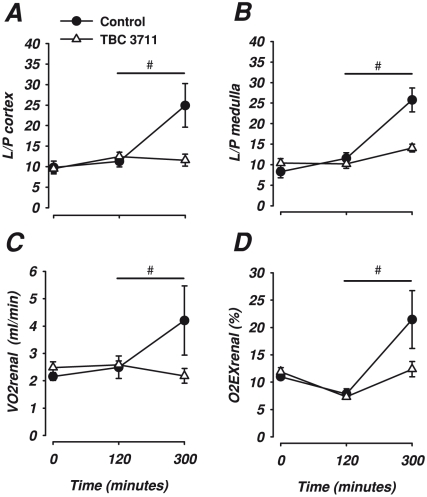
Renal oxygen transport and metabolism. Changes in lactate/pyruvate ratio cortex (L/P cortex) (A), lactate/pyruvate ratio medulla (L/P medulla) (B), Renal oxygen consumption (VO2renal) (C) and Renal oxygen extraction (O2EXrenal) (D) in response to lipopolysaccharide infusion and treatment with either the selective ETA antagonist, TBC 3711 (2 mg·kg −1, n = 8) or control (n = 8) Data are expressed as mean ± SEM. # indicates a significant difference between TBC 3711 and control in response to lipopolysaccharide. (ANOVA repeated measures including 120, 180, 240 and 300).

Renal oxygen delivery, DO2_renal_, increased (p<0.001 Table 2), ERO2_renal_ decreased but renal oxygen uptake VO2_renal_ did not change significantly (Figure 3, for both). For the interstitial fluid in the renal cortex, glucose_cort_ decreased whereas lactate_cort_ and pyruvate_cort_ increased (p<0.001, for all three, Table 2). No significant change in cortical lactate to pyruvate ratio, L/P_cort_ was observed (Figure 3). In the medulla no changes in glucose_med_ or L/P_med_ ratio was observed, but a significant increase in both lactate_med_ and pyruvate_med_ was seen (p<0.001, for both Table 2).

### Effect of treatment (120–300)

#### Systemic parameters

Cardiac output continued to decrease throughout the experiment and treatment with TBC 3711 had no significant effect on this decline (Table 1). However, TBC 3711 reduced blood pressure significantly compared with the control group (p<0.001, Figure 2). Tachycardia was present in both groups and TBC 3711 treatment had no significant chronotropic effect (Table 1). Body temperature increased in both groups as well as arterial lactate (Table 1). TBC 3711 attenuated the decrease in pH (Table 1). ET-1 levels in arterial plasma and renal vein remained elevated (p = 0.001, Table 1) throughout the experiment. Systemic extraction ratio increased significantly as a result of a decreased oxygen delivery and increased oxygen uptake in both groups (data not shown).

#### Renal parameters

Renal artery blood flow decreased as well as renal vascular conductance with no significant effect of TBC 3711 (Figure 1 and Table 1, respectively). Laser Doppler flowmetry in the cortex decreased in both groups, but TBC 3711 abolished the decrease seen in the renal medulla (Figure 2). Renal vein lactate increased with no significant group difference (Table 2). Diuresis, creatinine clearance and fractional sodium excretion was considerably reduced in both groups (Figure 2). Renal oxygen delivery (Table 2) decreased to baseline levels in both groups but renal oxygen uptake and renal oxygen extraction ratio was significantly lower in the TBC 3711 group compared to control (Figure 3, for both). Interstitial fluid levels of glucose in the cortex decreased, but this was abolished by TBC 3711 (Table 2). Treatment with TBC 3711 also attenuated the increase in cortical interstitial fluid levels of lactate and as a result interstitial fluid lactate/pyruvate (L/P) ratio was lower in the TBC 3711 treated group (Table 2 and Figure 3). Cortical pyruvate levels increased as a response to TBC3711 but were unchanged in the control group. No significant change in interstitial fluid glucose but an increase in lactate in both groups was seen in the medulla (Table 2, p<0.05 for both). However animals treated with TBC 3711 had a significant increase in pyruvate, thus generating a significantly lower L/P-ratio in the interstitial fluid (Table 2 and Figure 3).

## Discussion

The present study aimed to investigate the role of endothelin in mediating renal microcirculatory impairment through ETA activation in endotoxin induced acute kidney injury. The main finding was that treatment with an ETA-antagonist attenuated the decline in renal medullary perfusion, which was also reflected by signs of reduced ischemia in the medulla. This effect was independent of changes in total renal blood flow and was not a result of increased arterial blood pressure, suggesting that the effect was mediated on a microcirculatory level. Furthermore, ETA-receptor antagonism also attenuated renal oxygen extraction and the L/P-ratio measured in the renal cortex, but had no significant effect on diuresis or creatinine clearance.

Ischemia due to renal vasoconstriction is a plausible cause of septic acute kidney injury [1], although other mechanisms have been postulated as well [19]. Previously we have shown that treatment with the dual ETA/ETB antagonist tezosentan attenuates the decrease in total renal blood flow and renal cortical microcirculation caused by endotoxemia [16]. These results were not reproduced here. A reason for this may be that the effect on cardiac index by the ETA-antagonist in this study was less pronounced than the effect of tezosentan in the previous investigation. Here we used TBC 3711, a highly selective ETA antagonist with a selectivity of 441,000 fold for ETA over ETB. It has previously been used to investigate ETA-mediated mechanisms in pigs [7], [20], [21], including a recent report concerning microcirculatory derangements in the gut during endotoxemia [7]. The dose of TBC 3711 used was chosen based on previous experiments [7] and had no effect on plasma ET-1 levels. The clearance of ET-1 from the circulation is suggested to be mediated mainly by ETB receptors, and inhibition of ETB leads to increasing levels of ET-1 in the circulation [22]. Thus, it appears that no ETB antagonism was present after TBC 3711 administration in the current experiments. A group treated with a selective ETB-antagonist was not used as this has in previous experiments drastically reduced survival in endotoxemic pigs [23].

The current results suggest that endothelin reduces renal medullary perfusion in normotensive endotoxemic pigs due to activation of ETA-receptors. This is in contrast to previous findings in rats, where no change in medullary circulation was observed as an effect of treatment with the ETA antagonist BQ-123 in endotoxemia [24]. This discrepancy may reflect the fact that medullary microvascular flow was not reduced by lipopolysaccharide *per se* in that study (ref 24). Furthermore, we used a slightly longer observation period and as evident by Fig. 1D the reduction in medullary flow occurred after more than 2 hours of endotoxemia. The species difference could also be a factor since potential disparities in the distribution of endothelin receptors in the kidney between the rat and the pig may have functional implications.

The maintained medullary perfusion in the treatment group was not perfusion pressure dependent, since arterial pressure was significantly reduced by TBC 3711. Moreover, no difference in total renal blood flow was detected between groups indicating that the effect of TBC 3711 was on a microcirculatory level. Under normal physiological conditions intravenous infusion of ET-1 reduces medullary blood flow predominantly via ETA-activation [25]. This has been suggested to be due to a decrease in blood flow in the descending vasa recta [26], limiting the supply of blood to the medulla and shunting it to more superficially situated nephrons. The preservation of medullary microcirculation by ETA antagonism in this study may also in part be due to excessive stimulation of ETB when blocking ETA, thus promoting increased medullary vasodilatation by ETB via NO. On the other hand, increased ETB activity should have promoted other ETB mediated effects, such as changes in diuresis or renal sodium handling, which could not be detected in the present experiments.

In the early phase of endotoxemia, oxygen delivery to the kidney was increased, indicating that oxygen supply was not impaired at the onset of renal damage. ETA antagonism was associated with a reduced total renal oxygen extraction. As we measured *total* renal oxygen delivery and consumption, we cannot conclude if this reduction was a local medullary effect or if the renal cortex was affected as well. The most obvious explanation for a difference in renal oxygen consumption is a discrepancy in reabsorption of sodium, as this is the most oxygen demanding process in the kidney. However, there were no differences in sodium handling between the groups that could explain the difference in renal VO_2_. The natriuretic effect of ET-1 is also assumed to be mediated largely through activation of ETB [13]. Furthermore, there were no changes in creatinine clearance or renal blood flow which may also affect renal VO_2_. However, the control animals developed a more significant acidosis compared to TBC 3711-treated pigs, which may influence renal oxygen handling [27]. Lower pH (or higher carbon dioxide) reduces the binding affinity of oxygen to hemoglobin (Bohr effect [28]) and this will result in more oxygen being released in the kidney when metabolic demand increases. This effect has been highlighted in a rat model [29], but the relative impact in the porcine kidney remains to be explored.

Though no significant change in renal vein lactate was found in these experiments, local interstitial lactate-differences in cortex and medulla was observed with the use of microdialysis catheters. Analysis of interstitial fluid with microdialysis makes it possible to gain information about local metabolic changes and have been used previously in endotoxemia [30]. Lactate, pyruvate as well as the lactate/pyruvate ratio increased in both cortex and the medulla in response to endotoxin in the control group, but not in animals treated with TBC 3711. An elevated L/P ratio is usually interpreted as a sign of anaerobic metabolism. As renal perfusion decreased in control animals, ischemia is a likely cause of this effect in the medulla. However, aerobic glycolysis also leads to increased lactate levels without hypoxia in sepsis [31]. This non-anaerobic mechanism for increased lactate may, in part, be an explanation to our findings in the renal cortex where ETA-antagonism attenuated the increase in L/P ratio, but had no apparent effect on the microcirculatory blood flow. There was also a change in metabolism in the cortex, as indicated by reduced glucose levels and increased lactate levels. TBC 3711 attenuated the increase in lactate and the decrease in glucose but caused a significant increase in pyruvate, indicating a preserved gluconeogenesis converting lactate to pyruvate and subsequently glucose. Thus, disturbed oxidative metabolism is, at least partly, a plausible explanation for endotoxemia induced increase in L/P-ratio in the renal cortex, and this may be improved by ETA-receptor antagonism. However, further studies evaluating mitochondrial function or ATP levels in renal tissue are needed to investigate this hypothesis.

The hypothesis that selective ETA antagonism would improve diuresis by preserving ETB function was not supported by the experimental results. This may be due to an inadequate observation period, but may also suggest that the relative importance of using a specific ETA-antagonist, and thus not inhibiting ETB, for maintaining diuresis during endotoxemia is limited.

It is relevant to point out some limitations of this study. First, this study was performed in anaesthetized animals and anaesthesia *per se* has the potential to impair renal function in endotoxemia [32]. However, the extensive and invasive methods used for renal monitoring made general anesthesia necessary. No sham animals, i.e. surgically instrumented pigs followed for 5 hours but without endotoxin-infusion, were included in the study. It is reasonable to assume that surgery *per se* had an effect on some of the measured variables herein. This is a potential limitation as it is impossible to entirely rule out that ETA-antagonism attenuated a reduction in renal microcirculation caused by surgery. However, we find this unlikely as a study using a 22-hour porcine model of endotoxemia with similar instrumentation as the present investigation could not demonstrate an effect of surgery [33]. Furthermore, in prior studies by us in pigs, using the same degree of endotoxemia, the response in renal hemodynamics and renal function was similar although the renal instrumentation was less extensive compared to the present investigation [34], [35]. Another limitation is that urine production and creatinine clearance, as well as the parameters calculated from those measurements, reflects the function of both kidneys but all measurements of renal hemodynamics and renal metabolism were performed only on the left kidney. In addition, there are limitations to the Laser Doppler flowmetry method, which measures red blood cell velocity rather than quantitative blood flow. This method of microcirculatory monitoring is also unable to detect any heterogeneity in the renal microcirculation.

We conclude that TBC 3711, a selective ETA antagonist, attenuates endotoxemia induced microcirculatory impairment and ischemia in the renal medulla. Reduced total renal oxygen extraction and cortical L/P-ratio by TBC 3711, without effects on cortical blood flow, further suggest additional metabolic effects of ETA activation. However, in the short-term perspective, selective ETA-antagonism does not improve renal function in this model of endotoxin induced acute kidney injury.
